# No Escaping the Rat Race: Simulated Night Shift Work Alters the Time-of-Day Variation in BMAL1 Translational Activity in the Prefrontal Cortex

**DOI:** 10.3389/fncir.2017.00070

**Published:** 2017-10-04

**Authors:** Andrea R. Marti, Sudarshan Patil, Jelena Mrdalj, Peter Meerlo, Silje Skrede, Ståle Pallesen, Torhild T. Pedersen, Clive R. Bramham, Janne Grønli

**Affiliations:** ^1^Bergen Stress and Sleep Group, Department of Biological and Medical Psychology, University of Bergen, Bergen, Norway; ^2^Department of Biomedicine, University of Bergen, Bergen, Norway; ^3^KG Jebsen Centre for Neuropsychiatric Disorders, University of Bergen, Bergen, Norway; ^4^Groningen Institute for Evolutionary Life Sciences, University of Groningen, Groningen, Netherlands; ^5^Dr. Einar Martens Research Group for Biological Psychiatry, Center for Medical Genetics and Molecular Medicine, Haukeland University Hospital, Bergen, Norway; ^6^Section of Clinical Pharmacology, Laboratory of Clinical Biochemistry, Haukeland University Hospital, Bergen, Norway; ^7^Department of Psychosocial Science, University of Bergen, Bergen, Norway

**Keywords:** circadian rhythms, sleep deprivation, cognition, synaptic plasticity, protein synthesis, eIF4E, BMAL1, arc

## Abstract

Millions of people worldwide work during the night, resulting in disturbed circadian rhythms and sleep loss. This may cause deficits in cognitive functions, impaired alertness and increased risk of errors and accidents. Disturbed circadian rhythmicity resulting from night shift work could impair brain function and cognition through disrupted synthesis of proteins involved in synaptic plasticity and neuronal function. Recently, the circadian transcription factor brain-and-muscle arnt-like protein 1 (BMAL1) has been identified as a promoter of mRNA translation initiation, the most highly regulated step in protein synthesis, through binding to the mRNA “cap”. In this study we investigated the effects of simulated shift work on protein synthesis markers. Male rats (*n* = 40) were exposed to forced activity, either in their rest phase (simulated night shift work) or in their active phase (simulated day shift work) for 3 days. Following the third work shift, experimental animals and time-matched undisturbed controls were euthanized (rest work at ZT12; active work at ZT0). Tissue lysates from two brain regions (prefrontal cortex, PFC and hippocampus) implicated in cognition and sleep loss, were analyzed with m^7^GTP (cap) pull-down to examine time-of-day variation and effects of simulated shift work on cap-bound protein translation. The results show time-of-day variation of protein synthesis markers in PFC, with increased protein synthesis at ZT12. In the hippocampus there was little difference between ZT0 and ZT12. Active phase work did not induce statistically significant changes in protein synthesis markers at ZT0 compared to time-matched undisturbed controls. Rest work, however, resulted in distinct brain-region specific changes of protein synthesis markers compared to time-matched controls at ZT12. While no changes were observed in the hippocampus, phosphorylation of cap-bound BMAL1 and its regulator S6 kinase beta-1 (S6K1) was significantly reduced in the PFC, together with significant reduction in the synaptic plasticity associated protein activity-regulatedcytoskeleton-associated protein (Arc). Our results indicate considerable time-of-day and brain-region specific variation in cap-dependent translation initiation. We concludethat simulated night shift work in rats disrupts the pathways regulating the circadian component of the translation of mRNA in the PFC, and that this may partly explain impaired waking function during night shift work.

## Introduction

Millions of people worldwide work at times that overlap with the normal time for sleep, resulting in significant cognitive impairment and somatic symptoms (Rajaratnam and Arendt, 2001). In humans, night shift work in both real-life and laboratory simulations, is known to induce circadian rhythm disturbance which has been linked to deficits in cognitive functions (Folkard, 2008; Veddeng et al., 2014; Maltese et al., 2016; Pilcher et al., 2016). Night workers often report impaired alertness and performance on duty, and the risk of errors and accidents is increased at night (Folkard and Tucker, 2003; Åkerstedt et al., 2010; Øyane et al., 2013; Kazemi et al., 2016).

Sleep is regulated by both a homeostatic regulated sleep propensity process that builds up across hours of wakefulness, and an independent circadian process that oscillates with a period of about 24 h. In humans, the circadian factor typically promotes sleep at night and wakefulness during the day (Borbély, 1982). Hence, night shift work induces a mismatch between work demands and the brain’s state promoting sleep due to the homeostatic and circadian sleep drive (Gupta and Pati, 1994; Flo et al., 2012). It is conceivable that night work and other forms of circadian rhythm disruption decrease alertness and cognitive functioning through disruption of processes involved in regulating synaptic strength and neuronal communication. Plasticity at the level of the synapse represents a set of dynamic changes in the strength of information transfer between neurons, considered essential for processing and storage of information in the brain, and for the ability to adapt to and learn from the external environment. Many of the molecular events associated with long-term modification of synaptic strength are subject to circadian regulation (Gerstner and Yin, 2010), including synapse to nucleus signaling, neuronal activity-dependent gene transcription and protein synthesis.

**Figure 1:**
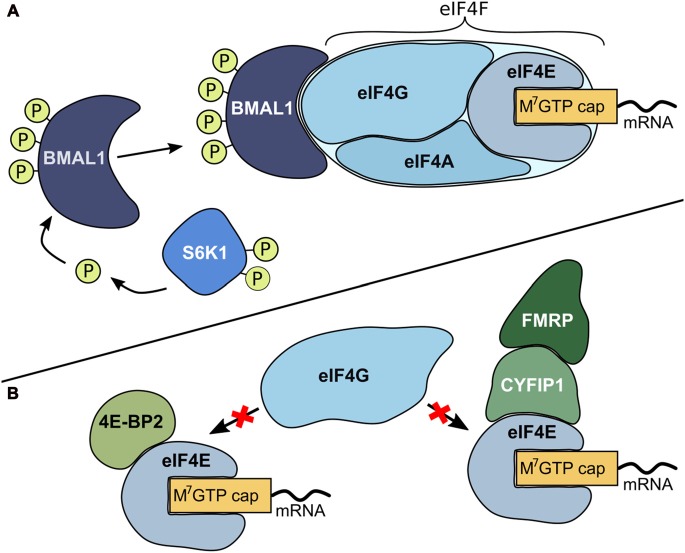
Mechanisms for the regulation of translation initiation. **(A)** Promotion of translation. Initiation starts with the formation of the eukaryotic initiation complex 4F (eIF4F). This complex consists of the translation initiation promoting factor eIF4E that binds to the mRNA m^7^GTP (cap) structure. Subsequently, eIF4E recruits cap-binding proteins like the scaffolding protein eIF4G and the RNA helicase eIF4A and form a multi-protein translation initiation complex. In its phosphorylated form, the circadian transcription factor brain-and-muscle arnt-like protein 1 (BMAL1) is capable of binding to the eIF4F complex and functions as a translation factor to further enhance translation. Ribosomal protein S6 kinase beta-1 (S6K1), an mTOR derived kinase, is a critical regulator for phosphorylation of BMAL1 (Lipton et al., 2015). **(B)** Repression of translation. There are several proteins that repress translation initiation. eIF4E-binding proteins (4E-BPs, for example 4E-BP2), cytoplasmic fragile X mental retardation-interacting protein (CYFIP1) and its binding partner fragile X mental retardation protein (FMRP) bind to eIF4E. When these proteins are bound to eIF4E, eIF4G cannot bind and hence eIF4F complex formation is prevented (Napoli et al., 2008; Bidinosti et al., 2010; Gkogkas et al., 2010; Gal-Ben-Ari et al., 2012; Kong and Lasko, 2012; De Rubeis et al., 2013).

Protein synthesis, synaptic plasticity and circadian rhythmicity are fundamental properties of the brain and are tightly regulated to environmental and cellular changes. The initiation step of mRNA translation is the most highly regulated step of protein synthesis (Siddiqui and Sonenberg, 2015; Bramham et al., 2016; Figure 1). Recently, the circadian clock has been linked to translation initiation processes (Lipton et al., 2015). In its phosphorylated form, the well-known circadian transcription factor brain-and-muscle arnt-like protein 1 (BMAL1) promotes cap-dependent translation (Figure 1A). The mTOR/S6 kinase beta-1 (S6K1) pathway is a critical regulator for phosphorylation of BMAL1 necessary to facilitate both association with the translational machinery and stimulation of protein synthesis (Lipton et al., 2015). In addition to several proteins that promote translation in neurons, there are also proteins that repress translation initiation (Napoli et al., 2008; Bidinosti et al., 2010; Gkogkas et al., 2010; Gal-Ben-Ari et al., 2012; Kong and Lasko, 2012; De Rubeis et al., 2013; Figure 1B).

A number of other cellular components are in addition involved in mediating protein synthesis-dependent synaptic plasticity. One regulator of synaptic activation is the activity-regulated cytoskeleton-associated protein (Arc). Arc acts as a multifunctional activity-induced hub protein important for mediating and organizing long-term synaptic plasticity, with impact on learning, memory and behavior (Guzowski et al., 2000; Kelly and Deadwyler, 2003; Bramham et al., 2010; Nikolaienko et al., 2017). Arc regulation has been implicated in sleep, wakefulness and in circadian rhythm regulation (Nishimura et al., 2003; Cirelli et al., 2004; Thompson et al., 2010).

We have recently established a rat model of shift work, where rats are exposed to enforced ambulation in slowly rotating wheels for 8 h/day, either in their rest phase (“rest work”; to simulate night shift work) or in their active phase (“active work”; to simulate day shift work; Marti et al., 2016; Grønli et al., 2017). Using this model, we observed a progressive intrusion of spontaneous cortical slow waves and micro-sleeps during rest work across four consecutive days, which was not observed during active work (Grønli et al., 2017). Similar findings have been observed in human studies, and likely contribute to decreased alertness during the night shift (Torsvall and Åkerstedt, 1987; Torsvall et al., 1989). Importantly, in our study, the degraded waking state during rest work could not be explained by sleep loss alone. Across the 16 h opportunity to sleep after work, the total amount of sleep was not different between rest and active workers.

Considering the importance of protein synthesis for activity-dependent synaptic plasticity and homeostatic plasticity (synaptic scaling), and the role of the circadian protein BMAL1 in translation initiation, it is conceivable that disruption of translation initiation may contribute to the impaired waking function observed in night shift workers. Sleep is associated with enhanced rates of protein synthesis (Cirelli et al., 2004), and sleep deprivation has been shown to have differential effects on the protein synthesis in the hippocampus and the PFC, respectively (for review see Grønli et al., 2014). Overall, insufficient sleep negatively affects protein translation, but sleep of good quality prior to sleep restriction may diminish these negative effects (Grønli et al., 2012). However, the effects of degraded waking function, or of simulated night shift work, on protein synthesis have not yet been considered. Moreover, it is still not known how other proteins involved in cap-dependent translation are regulated at different times of day and across different brain regions, or how simulated shift work affects these dynamics.

In the present study, we decided to use the cap-pulldown technique to determine the time-of-day and brain region specific variation in cap-dependent mRNA translation. Cap-pulldown is a valuable tool for examining the processes that are occurring directly on the mRNA cap, regulating the initiation of translation. It was decided to first characterize the time-of-day dependent variation in the expression of cap-associated proteins important for promoting translation initiation and repressing translation initiation, and regulation of synaptic plasticity. We hypothesized that cap-bound BMAL1, and its regulator S6K1, has specific time-of-day (lights ON vs. lights OFF) expression in two brain regions; the prefrontal cortex (PFC) and hippocampus, as these regions are implicated in cognition and are vulnerable to disturbance of circadian rhythms and sleep loss (Brown and Bowman, 2002; Cirelli et al., 2004; Gerstner and Yin, 2010; Karatsoreos et al., 2011; Alberca-Reina et al., 2015). Second, we wanted to investigate how these dynamics are affected by three consecutive days of simulated shift work.

## Materials and Methods

### Ethical Approval

This study was carried out in accordance with Norwegian laws and regulations, and The European Convention for the Protection of Vertebrate Animals used for Experimental and Other Scientific Purposes. The protocol was approved by the Norwegian Animal Research Authority (permit number: 2012463).

### Animals and Housing

Two batches of 40 male rats in total (*n* = 24 Wistar, nTach:WH; *n* = 16 Sprague-Dawley nTac:SD; Taconic, Silkeborg, Denmark) weighing approximately 300 g at arrival, were used in the study. Different rat strains were chosen because the supplier (Taconic) no longer deliver the Wistar strain. The procedures were otherwise the same for both experiments. All animals were group housed in individually ventilated cages (IVC, Techniplast, Buggugitate, Italy, 75 air changes/h) type IV (480 × 375 × 210 mm, 1500 cm^2^). The animals were maintained on a 12 h light/12 h dark (LD) schedule with lights on at 06:00 (zeitgeber time 0; ZT0). Lights were gradually dimmed on and off over a period of 1 h (fully on at 07:00 and fully off at 19:00). Filtered water and food were available *ad libitum* throughout the experiment (rat and mouse No. 1, Special Diets Services, Witham, Essex, UK). During the experimental protocol, all animals were single housed (IVC cage type III, 425 × 266 × 185 mm, 800 cm^2^).

### Experimental Protocol

To simulate shift work, animals were exposed to forced activity for 8 h per day, centered either in the rats’ normal active phase (active work; ZT14–22; *n* = 10) or in the rats’ normal rest phase (rest work; ZT2–10, *n* = 10). Animals were placed in automatically rotating wheels (Rat Running Wheel, TSE running wheel system, Bad Homburg, Germany; 24 cm diameter; 3 rpm; 1440 revolutions or 1.086 km of linear distance per 8 h session). Food and water was available *ad libitum*. Rotating wheel, feeders and water bottles were cleaned after each work session with 5% ethanol solution. Between sessions, animals were housed in their home cage. Work schedules were repeated for 3 days.

### Tissue Collection

Following the third work session, animals were placed in their home cage for 2 h. Subsequently, they were anesthetized with isoflurane, and sacrificed by decapitation. Active workers were sacrificed at ZT0, at lights ON, and rest workers at ZT12, at lights OFF. A separate group of undisturbed animals never exposed to simulated work were used to investigate time-point specific protein variation, and sacrificed at the same times as experimental animals (ZT0, at lights ON, *n* = 10; and ZT12, at lights OFF, *n* = 10).

### m^7^GTP (Cap) Pull-Down

m^7^GTP pull-down assays have been described in detail elsewhere (Panja et al., 2014). Bilateral hippocampus and PFC were separately homogenized in 1000 μl of m^7^GTP lysis buffer (50 mM Tris, 100 mM NaCL, 1 mM EDTA, NP-40 0.5%, 1 mM dithiothreitol, 1 mM Na_3_VO_4_, 50 mM NaF, and 1× protease inhibitor cocktail from Roche). The homogenate was centrifuged 10 min at 14,000 *g* at 4°C. For the m^7^GTP pull down, 300–400 μg of protein together with 30 μl of 7-methyl GTP-agarose beads (Jena bioscience #AC-141) were incubated 2 h at 4°C. Beads were washed three times with m^7^GTP lysis buffer and bound proteins were separated to an SDS-PAGE (10% gels). Immunoblotting was carried out as described above.

### SDS-PAGE and Immunoblotting

Antibodies used for immunoblotting were as follows: p-eIF4E (1:1000, Cell Signaling #9741), eIF4E (1:1000, Cell Signaling #9742), eIF4G (1:1000, Cell Signaling #2498), p-BMAL1 (1:1000, Cell Signaling #13936), total BMAL1 (1:500; Santa Cruz Biotechnology #sc365645), p-pS6k (1:1000, Santa Cruz Biotechnology #sc-7984), pS6k (1:1000, Sigma #SAB4502691), 4E-BP2 (1:1000, Cell Signaling #2845), CYFIP1 (1:1000, Upstate #07-531), fragile X mental retardation protein (FMRP; 1:1000, Abcam #17722), Arc (1:500; Santa Cruz Biotechnology #sc17839), and GAPDH (1:5000, Santa Cruz Biotechnology #sc32233).

Samples from cap pull-down assays and lysates were boiled in laemmli sample buffer (Bio-Rad) and resolved in 10% SDS/PAGE gels. Proteins were transferred to nitrocellulose membranes (Biorad, #162-0112) which were then blocked with 5% non-fat dry milk, probed with antibodies and developed using chemiluminescence reagents (Pierce, #32106). The blots were scanned using Gel DOC XRS+ (BIO RAD) and densitometric analyses were performed with ImageJ software (NIH, Bethesda, MD, USA). Blots treated with phospho-specific antibody stripped with 100 mM 2-mercaptoethanol, 2% SDS and 62.5 mM Tris-HCl, pH 6.8 at 50°C for 30 min, washed, blocked and reprobed with antibody recognizing total protein. Densitometric values expressed per unit of protein (tot-eIF4E/GAPDH as specified) applied to the gel lane. The cap-pulldown was conducted in order to assess changes in the association of eIF4E binding proteins with eIF4E. For this analysis the amount of eIF4G, p-BMAL1, total-BMAL1, CYFIP1, FMRP and 4E-BP2 was normalized to the total amount of recovered eIF4E. The phospho-proteins were normalized relative to the total protein on the same lane. Total proteins were normalized to loading control.

### Statistical Analyses

Significant effects of the time-of-day, work conditions, brain regions and experiments were determined using 2 × 2 factorial analysis of covariance (ANCOVA), with “time-of-day” or work condition (2 levels) and “brain region” (2 levels) as independent variables, and experiment as covariate. Different degrees of freedom in the results section are due to exclusion of some animals from analysis when showing values >2 standard deviations from the group mean. Additionally, one sample was lost due to a technical issue (ZT12 control, PFC). The *p*-value for significance was 0.05. Fisher’s LSD was applied as *post hoc* test. Cohen’s d was calculated as measure of effect size. As a mean for interpreting d, 0.2 is considered small, 0.5 medium and 0.8 large effect size, respectively (Cohen, 1988).

## Results

Changes in protein were assessed in homogenate samples obtained from micro-dissected PFC and hippocampus. Proteins actively bound to the cap-structure (cap-bound) and total concentrations of individual proteins available in the sample (input data) were measured. All statistical results are provided in Supplementary Tables S1–S6, and Supplementary Figures S1, S2 illustrate the results on input data.

### Time-of-Day Specific Variation in Different Brain-Regions

**Figure 2:**
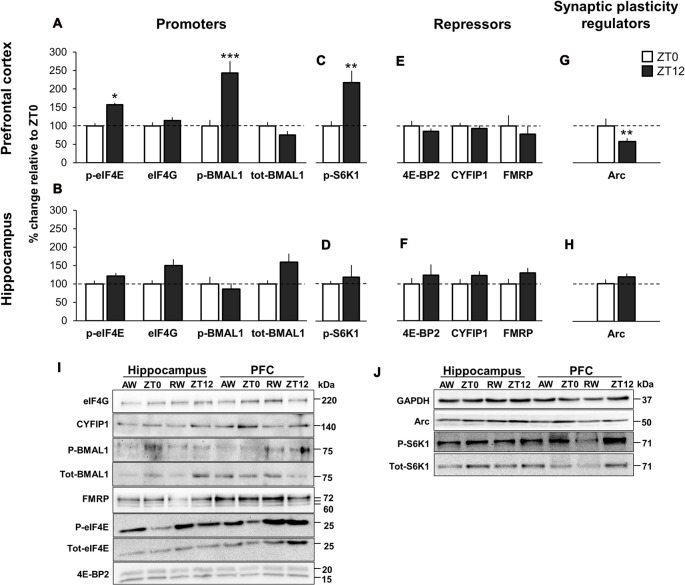
Time-of-day variation in promoters and repressors of cap-dependent translation initiation, and synaptic plasticity regulators in undisturbed animals. **(A,E)** m^7^GTP pull-down analysis of prefrontal cortex (PFC) and **(B,F)** hippocampus lysates. **(C,G)** Western blot analysis of PFC and **(D,H)** hippocampus lysates. Brain tissue was collected at lights ON (zeitgeber time 0, ZT0) and at lights OFF (ZT12). Quantification of immunoblot is expressed as percentage change relative to ZT0 (normalized to 100%). Error bars represent SEM. Significant differences: *p < 0.05; **p < 0.01; ***p < 0.001. **(I)** Representative immunoblot for **(A,B,E,F)**. Blots normalized to total eIF4E in the corresponding immunoblot. **(J)** Representative immunoblot for **(C,D,G,H)**. Blots normalized to GAPDH in the corresponding immunoblot.

We first examined expression levels of cap-bound promoters of translation. In PFC, two key translational promoters, the translation initiation factor p-eIF4E and the circadian transcription factor p-BMAL1 exhibited a significant effect of time-of-day, with significant increase of cap-bound phosphorylation at ZT12 (lights OFF) compared to ZT0 (lights ON; p-eIF4E: +57.7%, *p* = 0.03, *d* = 1.44; p-BMAL1: +143.4% at ZT12, *p* < 0.001, *d* = 1.57). This effect was not evident in the hippocampus (Figures 2A,B, immunoblots Figure 2I). The eIF4G, a scaffolding protein critical for formation of the translation initiation complex, and tot-BMAL1 (both phosphorylated and unphosphorylated BMAL1) showed no significant time-of-day effect, neither in the PFC nor in the hippocampus. Analysis of tissue lysate (inputs) suggests that all promoters were similarly expressed across both time points and brain regions (Supplementary Figures S1A,B).

Critical for BMAL1 to interact with the translational machinery is the regulator S6K1, as Lipton et al. (2015) showed that p-S6K1 phosphorylates BMAL1. In parallel with increased p-BMAL1 in PFC, p-S6K1 showed significantly higher expression at ZT12 compared to ZT0 (+118.1%, *p* = 0.002, *d* = 1.08). There was no significant effect of time-of-day in the hippocampus (+18.0%, *d* = 0.29; Figures 2C,D, immunoblots Figure 2J).

There were no significant time-of-day effects of the cap-bound translational repressors 4E-BP2, CYFIP1 and its binding partner FMRP (Figures 2E,F, immunoblots Figure 2I). Input data showed that total protein for all repressors were similarly available in all tissues and time points (Supplementary Figures S1C,D, Supplementary Table S4).

In PFC, the expression of Arc protein, a key regulator of long-term synaptic plasticity and synaptic scaling (Bramham et al., 2008, 2010; Korb and Finkbeiner, 2011; Shepherd and Bear, 2011) was decreased at ZT12 compared to ZT0 (−42.1%, *p* = 0.001, *d* = 1.13). There was no significant effect of time-of-day in the hippocampus (+18.3%, *p* = 0.68, *d* = 0.30; Figures 2G,H, immunoblots Figure 2J).

Overall, these results conform to findings by Lipton et al. (2015), suggesting that together with the known translational initiation promoter eIF4E, translation is linked to circadian timing by cap-bound p-BMAL1 at ZT12. Furthermore, the parallel increase in phosphorylated S6K1 at ZT12 supports Lipton et al. (2015) conclusions that BMAL1 is a substrate of S6K1. However, our data suggests that this is only the case in PFC and not in the hippocampus. Additionally, the present data suggest a differential role of Arc depending on time-of-day and brain region.

### Effects of Shift Work

To investigate the effects of three consecutive days of simulated shift work in either the normal active phase (“active work”; simulated day shift work) or the normal rest phase (“rest work”; simulated night shift work), changes in the translational regulators in the two groups were each compared to respective time-matched undisturbed controls (active work at ZT0; rest work at ZT12).

#### Active Work Does Not Significantly Affect Cap-Bound Translation Initiation

**Figure 3:**
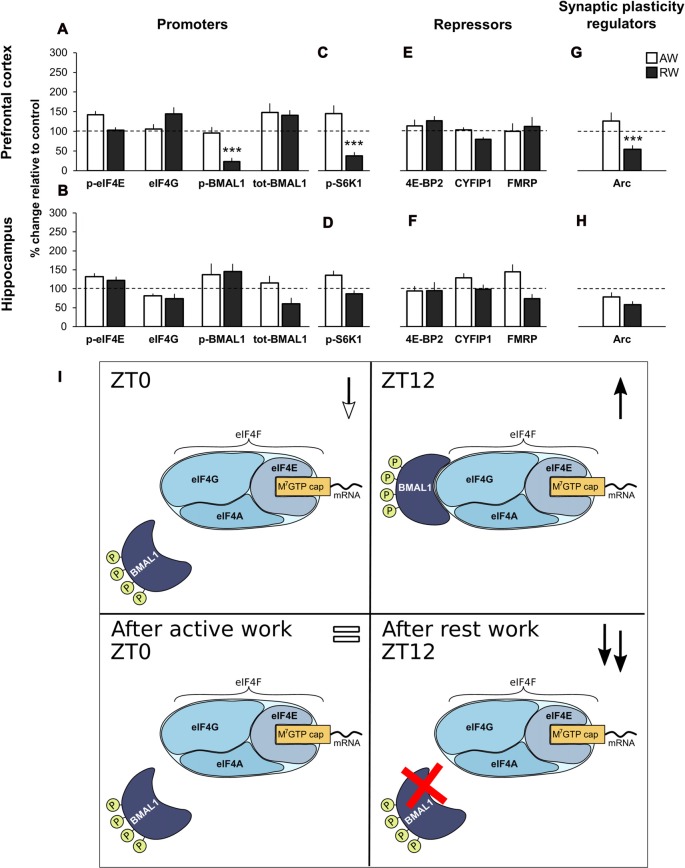
Effect of simulated shift work on promoters and repressors of cap-dependent translation initiation, and synaptic plasticity regulators relative to undisturbed controls. **(A,E)** m^7^GTP pull-down analysis of PFC and **(B,F)** hippocampus lysates. **(C,G)** Western blot analysis of PFC and **(D,H)** hippocampus lysates. Rats were exposed to forced activity during the active phase (AW, active work; brain tissue was collected at ZT0) or during the rest phase (RW, rest work; brain tissue was collected at ZT12). Quantification of immunoblot is expressed as percentage change relative to time-matched undisturbed control (normalized to 100%). Error bars represent SEM. Significant differences: ***p < 0.001. For representative immunoblot see Figure 2. **(I)** Schematic model of promotion of translation initiation in undisturbed animals (ZT0 and ZT12), and following simulated shift work (active work and rest work). ↓ decreased expression compared to ZT12; ↑ increased expression compared to ZT0; = similar expression compared to ZT0; ↑↑ increased expression compared to ZT12.

There was no significant effect of active work on the expression of the translational promoters (p-eIF4E, eIF4G, p-BMAL1 and tot-BMAL1), neither for the cap-bound proteins, the input proteins, nor for the expression of phosphorylated S6K1. Effect sizes were small (>0.5) for all proteins measured, with the exception of p-eIF4E and p-S6K1 in PFC (+25.7%; *d* = 0.53 and +44.9%, *d* = 0.55, respectively). This is illustrated in Figures 3A–D (immunoblots Figure 2I).

There was also no significant change after active work on the expression of the repressors (4E-BP2, CYFIP1 and FMRP, cap-bound and input) or the expression of Arc (Figures 3E–H, immunoblots Figures 2I,J).

Input data showed that all promoters and repressor proteins were available similarly across groups (active work and time-matched control; Supplementary Figures S2A–D, immunoblots Supplementary Figure S1E).

These data indicate that 3 days of simulated day work does not significantly alter regulators of translation initiation, neither in the PFC nor hippocampus.

#### Rest Work Impairs Regulation of Translation Initiation in PFC by Reducing Cap-Bound p-BMAl1, p-S6K1 and Arc

Following rest work, the circadian translational promoter p-BMAL1 was significantly reduced in PFC (−77.0%, *p* < 0.001, *d* = 1.97), as was its regulator p-S6K1 (−62.4%, *p* < 0.001, *d* = 1.29). There was no significant effect of rest work in terms of the expression of cap-bound phosphorylated eIF4E and eIF4G (Figures 3A–D). There was no significant effect of rest work in the hippocampus.

No significant effect of rest work was found on the expression of the repressors (4E-BP2, CYFIP1 and FMRP, cap-bound and input; Figures 3E,F).

Input data showed that all promoters and repressor proteins were available similarly across groups (rest work and time-matched control) except for a significant increase in available p-eIF4E in the hippocampus following rest work (+107.2%, *p* = 0.004, *d* = 1.43; Supplementary Figures S2A–D, immunoblots Supplementary Figure S1E).

The synaptic plasticity regulator Arc was significantly reduced in PFC following rest work (−46.5%, *p* < 0.001, *d* = 1.49; Figures 3G,H).

According to our data, phosphorylated BMAL1 and p-S6K1 are normally higher in the PFC at ZT12 compared to ZT0. The present results showing a reduced p-BMAL1 and p-S6K1 after simulated night shift work demonstrate an impaired circadian promotion of translation initiation in PFC (Figure 3I).

### Effects of Experiment

Data included in the analyses was pooled from two separate experiments on separate rat strains, as described in the method section. Thus, ANCOVA with experiment as covariate was run to examine whether any significant differences would occur between experiments. Overall, there were no significant effects of experiment on the translational promoters eIF4G and p-BMAL, both cap-bound and input. An effect of experiment was present in some analyses on translational repressors but the variation between experiments did not affect the variation across time-of-day, groups or brain tissue (Supplementary Tables S1–S6). Considering the overall uniformity of the data across experiments, and that our main findings on p-BMAL1 was upheld across rat strains, these findings suggest that the changes in protein expression reflect functionally meaningful impacts of time-of-day and rest work.

## Discussion

The aim of the present study was to examine time-of-day and brain-region specific variation in cap-dependent translation initiation in undisturbed rats and in rats exposed to simulated shift work. Decreased alertness, cognitive performance and quality of wakefulness has been observed in both humans and animals following night shift work, however, the neurochemical basis for these disruptions had yet to be investigated.

We found time-of-day specific differences in cap-bound protein expression and phosphorylation state in the PFC, in both undisturbed rats and rats exposed to simulated shift work. In undisturbed control rats, an upregulation of translational promoters was observed at the end of the resting phase (ZT12) as compared to the end of the active phase (ZT0). No significant up- or downregulation of translation initiation processes in the hippocampus were observed at ZT12 compared to ZT0.

Forcing rats to work during their normal active phase (active work) only resulted in non-significant changes in markers of protein synthesis regulation compared to time-matched controls. In contrast, a 3-day protocol of forced work during the normal resting phase (rest work) resulted in decreased phosphorylation of the circadian clock protein and translational promoter BMAL1 and its regulator S6K1, as well as decreased expression of the synaptic plasticity associated protein Arc in PFC. There were no significant effects of either active work or rest work on translation regulators in hippocampus. These results indicate that while the pathways regulating the circadian component of protein synthesis in the hippocampus appears to be spared, a disruption in this pathway in the PFC may underlie the cognitive deficits observed during night shift work.

### Cap-Dependent Translation Initiation in Undisturbed Animals

The cap-pulldown technique is a valuable tool for examining changes in translation initiation activity, based on altered binding of translation factors to the 5′-cap structure and associated eIF4E. Previously, Lipton et al. (2015) used cap-pulldowns in mouse hepatic tissue to show diurnal regulation of translation mediated by phosphorylation of BMAL1, with binding of p-BMAL peaking during the active phase. The present study demonstrates time-of-day dependent variation in cap-bound regulators of translation in the brain. Within the PFC of undisturbed animals, we observed that the cap-bound translational promoters p-eIF4E and p-BMAL1 were significantly increased at ZT12 compared to ZT0. These results indicate that cap-bound p-BMAL1 is actively up-regulating translation initiation at ZT12 in PFC. Notably, the variation in the PFC was evident only in the cap-pulldown analysis, and not in terms of input protein. This indicates that diurnal changes in the abundance of specific translation factors on the cap is due to changes in binding activity, and not to changes in levels of translation factor expression. The result underscores the importance of examining the dynamics of translation factor binding, which is not revealed by immunoblotting of tissue lysates.

No significant up- or downregulation of cap-dependent translation were observed in hippocampus at ZT12 compared to ZT0. Diurnal variation in p-eIF4E in hippocampal lysates (peak at ZT6, and no change between ZT12 and ZT0) has been reported (Saraf et al., 2014), however changes in cap- binding were not measured in that study. Another study examined expression of input protein in the hippocampal proteome and found that 1.7% of proteins showed diurnal variation (Chiang et al., 2017). This does not amount to a large proportion, but among proteins showing circadian variation were several of those found in the mTOR signaling pathway, which regulates BMAL1 phosphorylation through S6K1 (Lipton et al., 2015; Chiang et al., 2017). In liver and brain cells *in vitro*, cytoplasmic BMAL1 regulates translation initiation through the mTOR complex (Lipton et al., 2015). Our results in PFC support this, as the increase in p-BMAL1 was mirrored by an increase in the mTOR-derived regulator p-S6K1.

The role of BMAL1 as a transcription factor in the cell nucleus, where it is involved in clock gene regulation, has been well-characterized (for review see Albrecht, 2012). However, the role of cytoplasmic BMAL1 protein seems to be far separated from its transcriptional role. In fact, mTOR is increasingly standing out as a key regulator of cytoplasmic BMAL1 function. Over-activation of mTOR through knockout of the tuberous sclerosis (TSC) gene in mice causes an increase in cytoplasmic BMAL1 protein, as well as decrease in BMAL1 degradation, without affecting transcription of BMAL1 (Lipton et al., 2017).

The role of BMAL1 and BMAL1-interacting proteins in the cytoplasm still remains largely uncharacterized (Lipton et al., 2015). Only a few studies have started to identify the role of BMAL1 as a cap-dependent promoter of translation. Our results demonstrate that time- and tissue-specific variations exist in cap-dependent translation, and that there is still much to elucidate about the role of BMAL1.

### Effect of Simulated Shift Work on Cap-Dependent Translation Initiation

Following rest work, we observed a reduction in cap-bound p-BMAL1 as well as its mTOR-stimulated regulator p-S6K1 in PFC. Importantly, we only observed changes to phosphorylation status of cap-bound BMAL1. There were no significant changes in cap-bound tot-BMAL1 or BMAL1 available as input protein. Thus, the observed changes seem to be directly related to dysregulation of the mTOR/S6K1 pathway in the PFC. These results suggest that the PFC may be particularly vulnerable to rest work. Although effect on PFC functioning after simulated night shift work has yet to be examined, other rodent models support that the PFC may be a brain region particularly vulnerable to disruption of circadian rhythms. In one study, exposure to a 10 h light/10 h dark cycle decreased neuronal complexity in the prelimbic PFC and impaired performance on the Morris water maze in mice (Karatsoreos et al., 2011). Importantly, reduction in performance was observed only when the task was sufficiently demanding and requiring cognitive flexibility, suggesting an important role for the PFC in this task.

Our data suggests that the mTOR/S6K1 pathway in the PFC is particularly vulnerable to night shift work at ZT12. Interestingly, both increased and decreased activity in mTOR signaling pathways have been shown to have detrimental effects on learning and memory processes (Lipton and Sahin, 2014). Two models reflecting over- and under- activity of mTOR (TSC mice and S6K1 knock out mice, respectively) both show deficits in the early stage of long-term potentiation (LTP) formation and in the acquisition of the Morris water maze test (Goorden et al., 2007; Antion et al., 2008). Additionally, expression of dominant-negative S6K1 mutant in rat medial PFC resulted in less active coping behavior in the forced swim test (Dwyer et al., 2015). It is tempting to speculate that disruption of these processes may contribute to the degraded waking state observed in rats exposed to simulated night shift work (Grønli et al., 2017).

Rest work did not impair the mTOR/S6K1-derived pathway regulating translation in hippocampus in the present study. Previous studies show mixed results when it comes to the impact of simulated night shift work or similar interventions on hippocampal functions. In one study, 5 weeks of rest work did not impair performance on a hippocampus-dependent instrumental learning task (Leenaars et al., 2012). It is possible that the hippocampus is more vulnerable to sleep loss than to circadian rhythm disturbance. The effects of sleep amount and quality on hippocampal cellular and molecular processes, critical for learning, memory and spatial navigation, have been well characterized (Meerlo et al., 2009; Prince and Abel, 2013; Havekes and Abel, 2017). In our model, total sleep time was reduced similarly in rest workers and active workers (11%–12% compared to baseline), and cumulative slow-wave energy during non-REM sleep did not differ between the groups (Grønli et al., 2017). Still, slow wave intrusion during work was observed in rest workers only. Moreover, mathematical modeling of the sleep state dynamics demonstrated that differences in slow-wave-sleep bout duration between active and rest work groups largely reflected a circadian effect (Rempe et al, unpublished data). Chronic phase shifting does disrupt hippocampal function (e.g., Craig and McDonald, 2008), and also causes a 10% reduction in sleep time in mice (Brager et al., 2013). If the hippocampus is sensitive to sleep deprivation specifically, our model may not have sufficiently sleep deprived the animals, and thus did not induce significant hippocampal effects.

Sleep loss due to forced activity may be affecting the neuroendocrine systems by altering the state or function of the hypothalamo-pituitary-adrenal (HPA) axis. Both the PFC and the hippocampus are brain regions sensitive to secretion of corticosterone. The hippocampus has a high concentration of glucocorticoid receptors and hence increased sensitivity to glucocorticoids as this brain region is involved in the negative feedback response to release of glucocorticoids. Corticosterone can affect clock gene regulation and synaptic function (Takahashi et al., 2013; Al-Safadi et al., 2014), thus we cannot rule out that the effects observed may be caused by increased activity in the HPA axis as a result of forced activity in the rest phase. However, since the present study demonstrates effects on translational modulators and markers of synaptic plasticity in PFC and not hippocampus, this suggests that simple effects of corticosterone alone are likely not an essential driver of the observed effects.

### The Synaptic Plasticity Regulator Arc; Diurnal Variation and Sleep

The role of Arc in synaptic plasticity and learning has been extensively studied, but its diurnal variation has been less examined. Arc expression has been shown to differ between time-points (night vs. day) in pineal cells (Yeung Lam et al., 2004), and is involved in circadian phase-shift in response to light in the SCN (Nishimura et al., 2003). In PFC, Arc mRNA shows diurnal variation, with higher expression at ZT1 vs. ZT11, indicating that transcription of Arc is regulated by a diurnal component in this brain structure (Calabrese et al., 2011).

Arc mRNA transcription typically increases during the active phase and decreases during the rest phase, throughout the cortex (Cirelli and Tononi, 1999; Cirelli et al., 2004; Grønli et al., 2014). However, translation of Arc protein does not necessarily follow the changes in transcription. In the present study, we showed that Arc protein was reduced at the end of rest phase (ZT12) relative to the end of active phase (ZT0) in PFC. These data mimic previous findings by Thompson et al. (2010). However, the present findings also indicate that Arc protein shows a brain-region specific expression. Arc protein was expressed in an opposite pattern in the PFC (highest at ZT0) compared to the hippocampus (highest at ZT12). Another study showed that Arc translation was in fact increased in the cat visual cortex during the early hours of sleep (2 h after sleep onset; Seibt et al., 2012). In a previous study, we showed that, while Arc mRNA was increased in PFC after 8 h sleep deprivation, Arc protein was not (Grønli et al., 2012). Thus, Arc protein expression appears to be dynamically regulated in a tissue-dependent way both by time-of-day, and by sleep and wake states (Grønli et al., 2014).

The effects of simulated shift work or disturbance of circadian rhythms on the dynamics of Arc expression, to our knowledge have not been investigated previously. The present study demonstrates that simulated night shift work in rats significantly reduced expression of Arc protein in the PFC at ZT12. Arc protein is most recognized as an activity-induced protein, and not commonly known to decrease from baseline levels. We believe this is an effect reflecting circadian disturbance, and not an effect of reduced sleep as the 8 h forced ambulation increases the daily time awake and reduces the daily time in sleep equally in both rest- and active workers (Grønli et al., 2017). The only difference between rest- and active workers was the time at which the simulated shift work occurred. Our results on reduced expression of Arc protein in the PFC following rest work further indicate that the PFC may be more vulnerable than the hippocampus to the effects of night shift work.

### Implications

The present work has provided a deeper understanding of the mechanisms that underlie the impact of shift work on protein synthesis, important for long-term modification of synaptic strength and the brain’s ability to adapt to and learn from the external environment. The present results suggest that the degraded wake functioning observed in both humans and animals, as well as in both field and laboratory studies, may derive from reduced synaptic efficiency in the PFC. The PFC is crucial for higher-order cognition and complex tasks, such as attention and decision making (Brown and Bowman, 2002). It was suggested already in 1993 that the PFC may be particularly vulnerable to the effects of sleep loss (Horne, 1993; Alhola and Polo-Kantola, 2007). We suggest that this vulnerability may not only be due to sleep loss, but rather due to a mismatch between work demands and the brain’s ability to overcome the homeostatic and circadian challenges imposed by night shift work. Future animal studies should combine behavioral testing, measures of wake functioning and measures of PFC protein synthesis to elucidate the possible underlying mechanisms.

One study that also examined the effects of simulated night work on the brain showed that SCN clock gene expression was unaffected (Salgado-Delgado et al., 2013). This is not surprising considering that the SCN is primarily synchronized to the environmental light/dark cycle, and light conditions remain unchanged in animal models of shift work utilizing forced activity (Salgado-Delgado et al., 2008; Granados-Fuentes and Herzog, 2013). However, changes in peripheral (hepatic) clock gene expression was reported, suggesting that simulated night shift work caused internal desynchrony, which may contribute to the negative metabolic effects often associated with night shift work (Salgado-Delgado et al., 2013; West and Bechtold, 2015; Marti et al., 2016). The present study indicates that internal desynchrony also occurs between brain regions, regulation of translation initiation in the PFC and hippocampus in this case. In light of previous and present results, simulated night shift work clearly has tissue-specific effects, which likely have effects on individual tissues, but may also affect entire networks more broadly through internal desynchrony. These effects largely remain to be elucidated.

The results of the present study also raise further questions. For example, are the observed changes in the mTOR/S6K1/BMAL1 pathway and changes in Arc interrelated or are these processes independent of one another? Additionally, the mTOR pathway has been implicated in regulation of both sleep and circadian rhythmicity. From a molecular biology standpoint the processes regulating sleep and wakefulness, and circadian timing, have for a long time been considered relatively separate. However, we are now beginning to see that many of the same pathways are implicated in both sleep and circadian processes. In the real world, sleep deficiency and circadian dysregulation often co-occur, as seen for example in shift workers (for review see Kecklund and Axelsson, 2016). It is time to start examining the complex interactive effects of sleep and circadian rhythm disturbance and their effects on bodily regulatory processes.

### Methodological Considerations

A few methodological aspects of this study deserve discussion. First, in the present study we used only two time-points of investigation, which precludes us from making any conclusions about the nature of circadian variation in cap-bound proteins. We used a 3-day work protocol, from this the questions emerge of whether the same effects would be observed after only 1 day of rest work; or consequently, if the protocol had been longer, would more detrimental effects or some form of adaptation occur. Second, some effects on translation may be compartmentalized to synaptic regions, which may go undetected in the present analysis of tissue lysate. Third, we have not investigated the potential mechanisms which may compensate for the negative effects of reduced cap-binding of certain initiation factors following rest work (Uniacke et al., 2012). In addition, one should consider that rats are nocturnal animals and may not respond to simulated shift work in the same way as a diurnal species would. However, our previous findings using this model suggest that effects observed in rats mimic effects described in humans (Marti et al., 2016; Grønli et al., 2017). Simulating shift work in ways which do not require forced activity (for review see Opperhuizen et al., 2015) may also provide information on the generalizability of the observed effects. Lastly, the experiment was carried out with two batches of rats, representing different strains. The ANCOVA suggested significant differences between experiments pertaining to translational repressors. However, our main findings on the translational promoters p-BMAL1 and p-S6K1 were similar across experiments/strains. The fact that the main results were upheld implies that these findings represent functionally meaningful impacts of night work.

## Conclusion

Based on the current findings, we conclude that simulated night shift work in rats disrupts the pathways regulating the circadian component of the translation of mRNA in the PFC. These disruptions may explain the degraded waking function observed in night shift workers. Future animal studies should take care to consider diurnal variations when investigating the effects of simulated night shift work on cognitive performance and pathways regulating protein synthesis and synaptic plasticity, as timing-dependent factors may well play a role in these processes. Studies merging behavioral testing and assessment of neurochemical consequences of shift work in animal models will aid in furthering our understanding of the molecular basis of cognitive performance.

## Author Contributions

JG, ARM, PM, StP, JM, TTP and CRB designed the study. ARM, JM, TTP and SS collected the data. ARM and SuP performed the biochemical analyses. ARM, JG and CRB performed the statistical analyses. All authors contributed to the interpretation of data and writing of the manuscript.

## Conflict of Interest Statement

The authors declare that the research was conducted in the absence of any commercial or financial relationships that could be construed as a potential conflict of interest.
