# Supplementary material for: No Escaping the Rat Race: Simulated Night Shift Work Alters the Time-of-Day Variation in BMAL1 Translational Activity in the Prefrontal Cortex
Source: Front Neural Circuits. 2017 Oct 4;11:70. doi: 10.3389/fncir.2017.00070 (PMC5649179; doi:10.3389/fncir.2017.00070)
Supplement: Supplementary file 1 [file Supplementary_Material.pdf]

Supplementary Material

No escaping the rat race: Simulated night shift work alters the time-of-day variation in BMAL1 translational activity in the prefrontal cortex

Andrea Rørvik Marti*, Sudarshan Patil, Jelena Mrdalj, Peter Meerlo, Silje Skrede, Ståle Pallesen, Torhild Thue Pedersen, Clive Bramham, Janne Grønli

*** Correspondence:** Andrea Rørvik Marti: andrea.marti@uib.no

1.1 Supplementary Figures

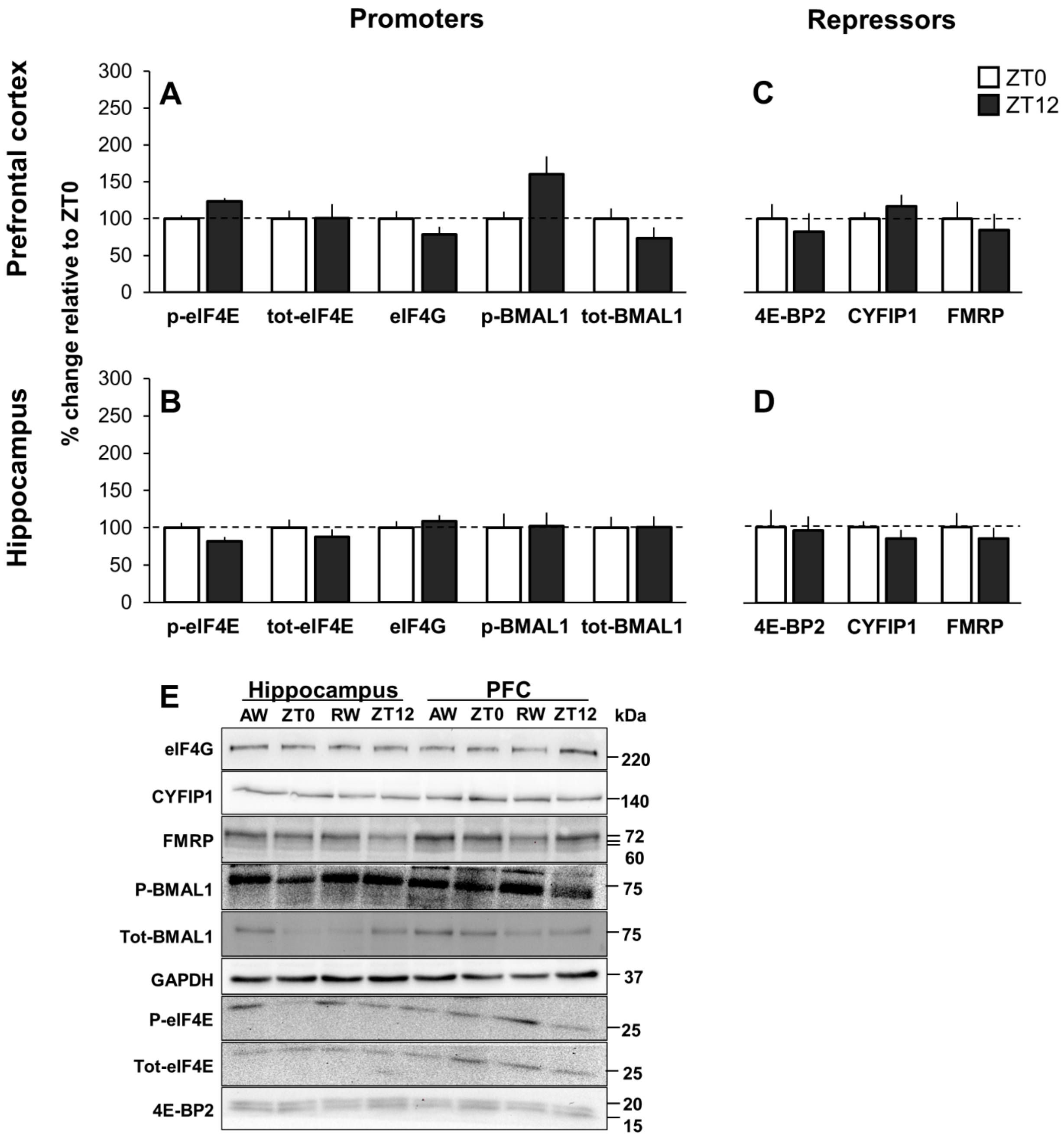

Supplementary figure 1. Time-of-day variation in promoters and repressors of cap-dependent translation initiation, and synaptic plasticity regulators in undisturbed animals. Western blot analysis of (**A, C**) prefrontal cortex and (**B, D**) hippocampus lysates. Brain tissue was collected at lights ON (ZT0) and at lights OFF (ZT12). Quantification of immunoblot is expressed as percentage change relative to ZT0 (normalized to 100%). Error bars represent SEM. (**E**) Representative immunoblot for (**A, B, C, D**). Blots normalized to GAPDH in the corresponding immunoblot.

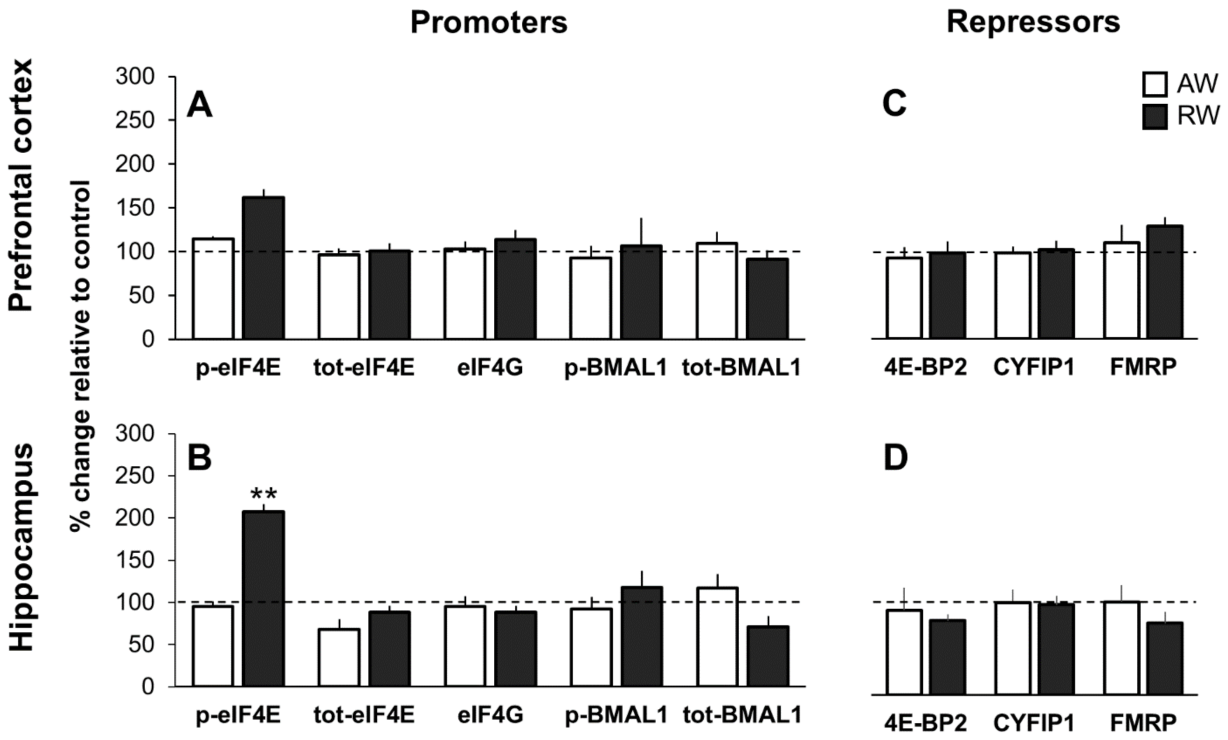

Supplementary figure 2. Effect of simulated shift work on promoters and repressors of cap-dependent translation initiation, and synaptic plasticity regulators relative to undisturbed controls. Western blot analysis of (**A, C**) prefrontal cortex and (**B, D**) hippocampus lysates. Rats were exposed to forced activity during the active phase (AW = active work, brain tissue was collected at ZT0) or during the rest phase (RW = rest work, brain tissue was collected at ZT12). Quantification of immunoblot is expressed as percentage change relative to time-matched undisturbed control (normalized to 100%). Error bars represent SEM. Significant differences: ** $p<.01$. For representative immunoblot see supplementary figure 1(E).

1.2 Supplementary tables

Supplementary table 1. Reported statistical main effects, analysis of covariance, time of day-analyses, M7GTP (cap) pulldown

		Time-of-day		Brain region		Time-of-day*brain region		Experiment	
**Protein**	**df**	**F**	**p**	**F**	**p**	**F**	**p**	**F**	**p**
p-eIF4E	1,32	5.01	**.03***	0.04	.85	1.12	.30	0.03	.86
eIF4G	1,34	2.03	.16	0.77	.39	0.60	.44	0.43	.52
p-BMAL1	1,34	5.11	**.03***	6.88	**.01****	7.70	**.009****	0.01	.95
Tot-BMAL1	1,34	0.78	.38	3.21	.08	3.22	.08	21.8	**<.001*****
4E-BP2	1,34	0.30	.59	9.94	**.003****	1.69	.20	10.1	**.003****
CYFIP1	1,33	0.18	.67	1.57	.22	0.48	.50	2.08	.16
FMRP	1,32	0.03	.87	4.83	**.03***	1.30	.26	26.8	**<.001*****

Significant differences: * p<.05; **p<.01; ***p<.001

Supplementary table 2. Reported statistical main effects, analysis of covariance, active work-analyses, M7GTP (cap) pulldown

		Active work		Brain region		Active work* brain region		Experiment	
**Protein**	**df**	**F**	**p**	**F**	**p**	**F**	**p**	**F**	**p**
p-eIF4E	1,34	2.40	.13	1.18	.29	0.01	.98	0.91	.35
eIF4G	1,35	0.06	.81	1.50	.23	1.84	.18	1.45	.24
p-BMAL1	1,35	0.40	.53	0.89	.35	0.62	.44	0.10	.76
Tot-BMAL1	1,35	1.34	.26	0.37	.55	0.37	.55	14.3	**<.001*****
4E-BP2	1,34	0.05	.83	2.47	.13	0.61	.44	7.81	**.008****
CYFIP1	1,33	0.65	.42	1.72	.20	0.54	.47	2.01	.17
FMRP	1,33	0.40	.53	6.14	**.02***	0.59	.45	24.4	**<.001*****

Significant differences: * p<.05; **p<.01; ***p<.001

Supplementary table 3. Reported statistical main effects, analysis of covariance, rest work-analyses, M7GTP (cap) pulldown

		Rest work		Brain region		Rest work* brain region		Experiment	
**Protein**	**df**	**F**	**p**	**F**	**p**	**F**	**p**	**F**	**p**
p-eIF4E	1,33	0.78	.38	0.06	.82	0.35	.56	4.71	**.04***
eIF4G	1,34	0.06	.81	0.01	.90	1.65	.21	3.80	.06
p-BMAL1	1,34	7.19	**.01****	2.12	.15	17.22	**<.001*****	0.26	.61
Tot-BMAL1	1,34	0.54	.47	1.75	.19	2.97	.09	17.4	**<.001*****
4E-BP2	1,34	0.07	.79	10.4	**.003****	0.54	.47	6.10	**.02***
CYFIP1	1,33	0.31	.58	6.94	**.01****	0.28	.60	1.42	.24
FMRP	1,32	0.22	.64	4.61	**.04***	0.73	.40	36.3	**<.001*****

Significant differences: * p<.05; **p<.01; ***p<.001

Supplementary table 4. Reported statistical main effects, analysis of covariance, time-of-day-analyses, inputs

Protein	df	Time-of-day		Brain region		Time-of-day* brain region		Experiment	
		**F**	**p**	**F**	**p**	**F**	**p**	**F**	**p**
p-eIF4E	1,32	0.01	.97	0.41	.53	1.51	.23	0.01	.96
Tot-eIF4E	1,34	0.17	.68	0.01	.92	0.17	.68	0.60	.44
eIF4G	1,32	0.62	.44	0.17	.68	2.47	.13	0.35	.56
p-BMAL1	1,33	2.76	.11	0.15	.70	2.32	.14	0.01	.91
Tot-BMAL1	1,33	0.91	.35	1.51	.23	0.98	.33	1.35	.25
4E-BP2	1,33	0.19	.66	1.19	.28	0.03	.86	3.33	.08
CYFIP1	1,33	0.01	.93	2.42	.13	1.51	.23	1.49	.23
FMRP	1,34	0.93	.34	2.82	.10	0.02	.88	0.18	.67
p-S6K1	1,31	8.31	**.007****	16.5	**<.001*****	5.42	**.03***	13.04	**.001*****
Arc	1,33	2.65	.11	6.47	**.02***	5.74	**.02***	2.19	.15

Significant differences: * p<.05; **p<.01; ***p<.001

Supplementary table 5. Reported statistical main effects, analysis of covariance, active work-analyses, inputs

Protein	df	Active work		Brain region		Active work* brain region		Experiment	
		**F**	**p**	**F**	**p**	**F**	**p**	**F**	**p**
p-eIF4E	1,33	0.04	.85	1.95	.17	0.27	.61	0.39	.54
Tot-eIF4E	1,35	0.06	.81	0.84	.37	0.26	.61	4.33	**.05***
eIF4G	1,31	0.01	.95	4.89	**.03***	0.13	.72	0.01	.91
p-BMAL1	1,33	0.22	.64	2.53	.12	0.01	.94	5.69	**.02***
Tot-BMAL1	1,33	0.46	.50	5.06	**.03***	0.003	.95	3.44	.07
4E-BP2	1,34	0.11	.75	0.96	.33	0.01	.96	1.99	.17
CYFIP1	1,34	0.01	.91	12.2	**.001*****	0.07	.80	3.77	.06
FMRP	1,35	0.15	.70	3.90	.06	0.02	.88	1.33	.26
p-S6K1	1,32	3.02	.09	6.28	**.02***	0.19	.67	15.4	**<.001*****
Arc	1,34	0.20	.66	27.8	**<.001*****	1.64	.21	**4.41**	**.04***

Significant differences: * p<.05; **p<.01; ***p<.001

Supplementary table 6. Reported statistical main effects, analysis of covariance, rest work-analyses, inputs

Protein	df	Rest work		Brain region		Rest work* brain region		Experiment	
		**F**	**p**	**F**	**p**	**F**	**p**	**F**	**p**
p-eIF4E	1,32	12.8	**.001*****	0.12	.73	0.66	.42	1.70	.20
Tot-eIF4E	1,34	1.36	.25	0.76	.39	0.07	.79	0.01	.91
eIF4G	1,34	0.01	.98	0.01	.98	1.40	.25	0.15	.70
p-BMAL1	1,34	0.41	.53	1.67	.21	0.09	.77	2.66	.11
Tot-BMAL1	1,34	1.19	.28	0.60	.44	0.28	.60	1.02	.32
4E-BP2	1,32	0.45	.51	1.52	.23	0.17	.69	2.67	.11
CYFIP1	1,33	0.01	.98	0.12	.73	0.01	.97	0.75	.39
FMRP	1,33	0.21	.65	14.2	**<.001*****	2.85	.10	1.25	.27
p-S6K1	1,31	12.1	**.002****	15.4	**<.001*****	8.79	**.006****	**17.5**	**<.001*****
Arc	1,32	12.4	**.001*****	0.01	.94	0.08	.78	7.87	**.008****

Significant differences: * p<.05; **p<.01; ***p<.001
